# Construction of the Enterococcal Strain Expressing Immunogenic Fragment of SARS-Cov-2 Virus

**DOI:** 10.3389/fphar.2021.807256

**Published:** 2022-01-05

**Authors:** Alexander Suvorov, Tatiana Gupalova, Yulia Desheva, Tatiana Kramskaya, Elena Bormotova, Irina Koroleva, Olga Kopteva, Galina Leontieva

**Affiliations:** Scientific and Educational Center, Molecular Bases of Interaction of Microorganisms and Human of the World-Class Research Center, Center for Personalized Medicine, FSBSI, IEM, Saint-Petersburg, Russia

**Keywords:** probiotic, enterococcus, probiotic-based vaccines, SARS-CoV-2, immune response, S protein

## Abstract

Contemporary SARS-Cov-2 pandemic, besides its dramatic global influence on the human race including health care systems, economies, and political decisions, opened a window for the global experiment with human vaccination employing novel injectable vaccines providing predominantly specific IgG response with little knowledge of their impact on the mucosal immunity. However, it is widely accepted that protection against the pathogens at the gates of the infection - on mucosal surfaces—predominantly rely on an IgA response. Some genetically modified bacteria, including probiotics, represent attractive vehicles for oral or nasal mucosal delivery of therapeutic molecules. Probiotic-based vaccines for mucous membranes are easy to produce in large quantities; they have low cost, provide quite a long T-cell memory, and gut IgA response to oral vaccines is highly synchronized and strongly oligoclonal. Here we present a study demonstrating construction of the novel SARS-Cov-2 vaccine candidate employing the gene fragment of S1 SARS-Cov-2 gene. This DNA fragment was inserted in frame into major pili protein gene with d2 domain of enterococcal operon encoding for pili. The DNA sequencing proved the presence of the insert in enterococcal genome. RNA transcription, immunoprecipitation, and immune electron microscopy with human sera obtained from the SARS-Cov-2 patients demonstrated expression of SARS-Cov-2 antigens in bacteria. Taken together the data obtained allowed considering this genetically modified probiotic strain as an interesting candidate for vaccine against SARS-Cov-2.

## Introduction

The onset of the SARS-Cov-2 pandemic required the urgent preventive measures to limit the spread of the virus has accelerated the development of antiviral vaccines. All COVID-19 vaccines which are currently approved or authorized in the United States (Pfizer-BioNTech/Comirnaty, Moderna, and Janssen [Johnson and Johnson]), China, European Union (Astra Zeneca) or Russian Federation (Sputnik V) are effective against COVID-19, including severe disease, hospitalization, and death. Present data suggest lower effectiveness of the present vaccines against confirmed infection and symptomatic disease caused by the Beta, Gamma, and Delta variants compared with the ancestral strain and Alpha variant (Agrawal et al., 2021; Björk et al., 2021; Gushchin et al., 2021; Harder, et al., 2021; Kow and Hasan, 2021; Shapiro et al., 2021; Yin et al., 2021). The majority of the vaccines on the market indifferently on vaccine making approach, rely on the needle injection of the vaccine hoping for the immune recognition of the viral antigens and for establishment of the cellular and adaptive immune responses to the pathogen in case of its appearance in the organism.

However, such approaches produce a weak immune response on the mucous membranes at the gate of the infection which is oral or gut mucosa allowing the virus to enter the organism. This makes it possible to spread the disease through a fully vaccinated population and provide the possibility of artificial induction of the appearance of viral variants under the pressure of the targeted immune response inflicted by the vaccination. These and some other problems of contemporary vaccination can be solved by mucosal vaccines against SARS-Cov-2 providing the first line of defense against the pathogen. Here we describe the construction and preliminary study of novel mucosal vaccine candidate with enterococcal probiotic as the vector for viral antigens providing immune recognition of SARS-Cov-2.

## Materials and Methods

### Bacterial Cultures

*Enterococcus faecium* L3 and *Escherichia coli* strains DH5α and M15 were obtained from the collection of the Institute of Experimental Medicine and used as the recipients for transformation. *E. coli* strains were grown in Luria Bertani (LB) medium (Oxoid, United States) at 37°C with constant shaking*. E. faecium* L3 and its derivatives were grown in Todd Hewitt Broth (THB) (HiMedia, India) at 37°C for 14 h. LB agar (Lennox L agar, Thermo Fisher Scientific) and *Enterococcus* Differential Agar Base (TITG Agar Base) (Himedia, India) without antibiotic and with 10 μg/ml of erythromycin were used as a solid medium for cultivation, bacterial quantification, and identification of *E. faecium* L3 and erythromycin-resistant enterococcal transformants. The bacteria *E. coli* М15 SarsS were cultured on Terrific broth in the presence of ampicillin (100mcg/ml) and kanamycin (25 mcg/ml).

### Genetic Engineering and Protein Studies

#### Cloning of the *sars*S Gene Fragment

A fragment of the *sarsS* gene of 512 bp was chemically synthesized and originally cloned into the vector plasmid DNA pAL2-T (Eurogen, Russia).

А fragment of the gene encoding the S-protein of the SARS-CoV-2 virus was obtained by polymerase chain reaction (PCR) using primers Cov1 и Cov2 with incorporated sites for restriction endonucleases *BamHI* and *SacI* and synthesized fragment of the *sarsS* gene encoding the fragment of S-protein of SARS-CoV-2.

The obtained DNA fragment *sarsS* was cloned using the expression plasmid pQE-30 (Qiagen, Hilden, Germany). Recombinant plasmid DNA pQE-sarsS and an expression strain of *E. coli* M15-SarsS were obtained after cloning of the PCR product.

#### Purification of Recombinant Protein SarsS

After expression in the recombinant *E. coli* M15-SarsS strain protein SarsS was purified under denaturing conditions. Briefly, the bacteria were cultured on Terrific broth in the presence of ampicillin (100 mcg/ml) and kanamycin (25 mcg/ml) until the late logarithmic growth phase (OD 600 = 0.7 ÷ 0.9). Then, the expression of the recombinant protein was induced by the addition of IPTG and the cells were cultured for another 4.5 h. The cells were harvested by centrifugation and the cell pellet was frozen at -70°C. The thawed precipitate was resuspended in the buffer A (8 M urea, 0.1 M Na2HPO4, 0.1 M NaH2PO4, pH = 8.0) and the cells were lysed completely by gentle vortexing for 1 h at room temperature. After removing a cell debris, the protein was purified from the supernatant by using Ni Sepharose. The protein Cov1S eluted from Ni Sepharose (Qiagen, Hilden, Germany), under denaturing conditions revealed a single 24.5 ± 0.5 KDa band by Coomassie brilliant blue staining after 12% SDS-PAGE. The purified protein was refolded using two-step dialysis against 3 M urea, 0.1 M Na2HPO4/NaOH, pH = 9.2 during 2 h and 0.4 M NaCl, 0.02 M Na2HPO4/NaOH, pH = 9.2 overnight at 6°C without stirring. The resulting protein SarsS was sterilized by filtration using Millipore disposable filters (0.45 microns) and stored at 6°C. The analysis of the pure recombinant protein SarsS by MALDI TOF/TOF (Bruker Daltonics, Germany) confirmed that its sequence corresponds to the part of the SARS-CoV-2 S protein (data not shown).

### Production of Recombinant *E. faecium* L3-SARS

#### Development and Cloning of a Fusion Gene *entF- sarsS*

The fusion gene *entF-sarsS* was generated using recombinant suicidal plasmid pentF*-pspf* that was made earlier for making live probiotic pneumococcal vaccine (Gupalova et al., 2018). Plasmid *pentF-pspf* was obtained by inserting *pspf* sequence between two separate gene fragments of the probiotic *Enterococcus faecium* L3. In order to make the plasmid *entF-sarsS*, *pentF-pspf* was digested with *NdeI* and *EcoRI* which were flanking *pspf* sequence. *pspf* sequence was replaced with the chemically synthesized fragment of the *sarsS* gene of (512 bp) which also carried sites for *Nde*I and *EcoR*I for convenience of subcloning.

Resultant plasmid DNA was transformed into the *E. coli* DH5α with selection of the transformants on LA plates with 500 μg/ml erythromycin. To identify the *E. coli* clones with desired plasmid p*entF-sarsS* DNA primers K1 and K2 corresponding to the *sars*S gene were used. (Table 1). Plasmid p*entF-sarsS* was isolated employing Mini-Prep kit (Qiagen, Hilden, Germany) and used for electro transformation of enterococci.

**TABLE 1:** List of oligonucleotide primers used.

Primers	Direction 5′-3′	Nucleotide sequence from 5′ to 3′	Purpose
K1	Forward	TTGCAT​ATGGGT​TTC​CAA​CCC​ACT^a^	Gene fragment flanking for making *sarsS*
K2	Reverse	GTAGAA​TTCGTT​GTT​ACA​TGT​TCA	Gene fragment flanking for making *sarsS*
B1	Forward	TGA​GTG​AAC​CAC​AGC​CAG​AA	Integration of the *pentF-sarsS* plasmid DNA into the *Enterococcus* in chromosomal DNA
Seq F	Forward	GGA​CAC​CAC​AAC​CAT​CGA​AG	Sequencing of the PCR product of *pentF-S1*
Cov1	Forward	AAGGA​TCCATA​CAT​ATG​GGT​TTC​C	Cloning a gene fragment *sarsS* for protein production
Cov2	Reverse	TGT​CGA​CGGAG​CTCGAA​TT	Cloning a gene fragment *sarsS* for protein production
A1	Forward	GCT​CTA​GAG​CCG​ATG​AGA​GCA​GCT​GGT​ATT​G	Determining the presence of inserts and a fragment of the *sarsS* gene in *Enterococcus*
D1	Reverse	CAA​CAG​GAT​CCA​AAG​CAT​CGT​TGG	Determining the presence of inserts and a fragment of the *sarsS* gene in *Enterococcus*
Dal 1	Forward	TTG​AGG​CAG​ACC​AGA​TTG​ACG	D-alanine-D-alanine ligase
Dal 2	Reverse	TAT​GAC​AGC​GAC​TCC​GAT​TCC	D-alanine-D-alanine ligase

a The underlined area in the nucleotide sequences correspond to restriction sites used for cloning.

#### Transformation of Enterococcus With an Integrative Plasmid p*entF-sarsS* by Electroporation

For electroporation, *E. faecium* L3 was grown in 3 ml of Todd-Hewitt medium (THB) (HiMedia, India) overnight at 37°C, inoculated (2%) in 50 ml of THB medium and grown to OD_600_ = 0.3. Resultant culture was cooled on ice, washed three times with cold double distilled water with 10% glycerol by centrifugation 3,500 *g* at 4°C. The cell pellet was suspended in 0.5 ml of 10% sterile glycerol solution, precipitated and suspended in 0.3 ml of the same solution. A total of 50 μl of cell suspension was added to the electroporation cuvettes with a distance of 1 mm between the electrodes at a voltage of 2100 V.

A total of 300 ng of the integrative plasmid *pentF-sarsS* was added to 50 mcl of cells. The optimal pulse duration was 4–5 ms. After the discharge, 1 ml of THB medium was added to the cuvette; the bacterial suspension was incubated for 1 h at 37°С and plated on THA with 10 μg/ml of erythromycin. The appearance of *E. faecium* L3-SARS transformants was monitored after 24 h.

#### Transcription of the *SarsS* Protein Gene Fragment Inserted in Bacterial DNA

The expression of mRNA was studied using real-time PCR (rRT-PCR) with reverse transcriptase using primers specific for the S-protein. Bacteria were grown in THB medium at 37°C for 18 h*. E. faecium* L3-SARS was cultivated with 5 μg/ml of erythromycin (Sigma, United States). Bacteria were washed three times in PBS by centrifugation at 3,500 rpm for 20 min and suspended in PBS. There was 10x concentrate used for mRNA analysis. Isolation of total RNA was carried out using the GeneJET RNA Purification Kit (Thermo Scientific, Waltham, United States). The isolated RNA was treated with 1 U/µl DNase (Invitrogen, Waltham, United States) after which one-step rRT-PCR was performed on a SFX96 thermocycler (BioRad, Hercules, United States) using HS-qPCR SYBR Blue master-mix (Biolabmix, Novosibirsk, Russia). *SarsS* specific primers K1 и K2 were used for analysis of SarsS protein gene expression and D-alanine-D-alanine ligase gene of *E. faecium* L3 as follows: Dal1и Dal2 as the normalizing gene.

### Immunological Analysis

#### Immunization Schemes and Antibodies

To evaluate recombinant SarsS protein immunogenicity, mice were injected s. c. on the back two times at 3-week intervals with the 20-µg dose of protein per each immunization. The protein in 0.2 ml of PBS was emulsified together with 0.1 of Imject Alum (Thermo Scientific, United States) before the injection. Blood samples were taken from the submaxillary vein on the specified days or mice were bled 3 weeks after the last injection. The blood samples were centrifuged at 1,500×g for 10 min, and the collected sera were stored at −20°C.

To evaluate recombinant SarsS protein immunogenicity in rabbits, animals were injected i. c. into the sides three times at 3-week intervals. Three rabbits were immunized with the 120-µg dose of protein in PBS together with Imject Alum (Thermo Scientific, United States) in the ratio of 1:1 and in total volume of 1.0 ml and two control rabbits were immunized with PBS only with Imject Alum. Blood samples were taken from the ear vein of rabbits before the experiment and on the specified days. The collected sera were prepared and stored as described above.

The study used serum/plasma samples remaining from routine clinical tests of COVID-19 patients admitted to Vsevolozhsk Clinical Interdistrict Hospital, Leningrad Region, Russian Federation during March-April 2020. Positive SARS-Cov-2 results were identified by licensed laboratory tests (Imbian, Russia). The study employing the human sera was approved by the Local Ethics committee of the FSBSI “IEM” (protocol 3/20 from May 06, 2020). After receiving the approval of the Ethics Committee, the sera were handed over to the researchers none of whom had access to personal data of patients. As this is a retrospective study, informed consent was not required.

Monoclonal antibodies against spike glycoprotein S1 Cat. number: ATMA10164Mo were purchased from AtaGenix laboratories (Wuhan, China).

#### Western Blotting

For Western blotting bacterial lysates or SarsS solution were incubated with Laemmle buffer in the presence of *β*-mercaptoethanol at 95 °C for 5 min, followed by SDS-electrophoresis and transferred to nitrocellulose membrane for blotting (Sigma-Aldrich, United States). The membranes were blocked by 3% skim milk (Fluka, Germany)/PBS with 0.05% Tween 20 and incubated at 37°C for 60 min with primary monoclonal Anti-SARS-CoV-2 (S1) antibody (CR3022) (Atagenix laboratories, China) diluted 1:1,000 at the same buffer or serum obtained from COVID-19 patients diluted 1:250 followed by 60 min incubation in 3% skim milk with horseradish peroxidase-conjugated anti-human secondary antibodies (1:2000, Sigma, United States). Immunoreactive bands were detected using TMB liquid substrate system for membranes (Sigma). Bacterial lysates or SarsS solution were incubated with Laemmle buffer in the presence of *β*-mercaptoethanol at 95°C for 5 min followed by SDS electrophoresis and transferred to nitrocellulose blotting membrane (Sigma-Aldrich). Membranes were blocked in 3% skim milk (Fluka, Germany)/PBS with 0.05% Tween 20 and incubated at 37°C for 60 min with Anti-SARS-CoV-2 (S1) (CR3022) primary monoclonal antibody (Atagenix laboratories, China) diluted 1:1,000 in the same buffer, or serum obtained from patients with COVID-19, diluted 1:250. Next, a 60-min incubation was carried out in 3% skim milk with secondary anti-human antibodies conjugated with horseradish peroxidase (1:2000, Sigma, United States). Immunoreactive bands were detected using the TMB Liquid Substrate Membrane System (Sigma, United States).

#### ELISA Assay

ELISA assay was done as described earlier (Gupalova. et al., 2019). Briefly, Maxisorb 96-well plates (Nunc; Denmark) were coated overnight at 4°C with 0.25 μg/ml of protein S in 0.1 M sodium carbonate buffer pH 9.3. A series of twofold dilutions of the sample (100 μl) was added to duplicate wells and incubated for 1 h at 37°C. Between the different stages, the plates were washed with blocking buffer (0.05% Tween-20 in PBS). The same buffer was used for serum and reagents dilution. HRP-labeled goat anti mouse IgA or IgG antibodies (Sigma) were added (100 μl/well). After incubation at 37°C for 1 h, the plates were developed with 100 μl/well TMB substrate (BD Bioscience). A color was detected after 20 min of incubation after stopping the reaction with 30 µl of 50% sulfuric acid. The endpoint ELISA titers were expressed as the highest dilution that yielded an optical density at 450 nm (OD450) greater than the mean OD450 plus 3 standard deviations of negative control wells.

#### Detection of the Surface Display of the Recombinant SarsS Protein on *E. faecium* Cells

To determine the localization of protein SarsS on the surface of bacteria, we compared the results of sandwich and competitive ELISA on biofilms of the original and modified *Enterococcus*. For this purpose, we used the human polyclonal sera containing IgG specific to the S1 protein of the SARS-CoV-2.

The procedure for preparing enterococcal biofilms was carried out according to (Tabenski et al., 2014). Biofilms of original *E. faecium* L3 or genetically modified *E. faecium*.

L3-SARS were grown in 96-well plates in sterile THB with 0.5% yeast extract by incubation at 37°C for 24 h. To reduce the nonspecific binding of human serum IgG to the *E. faecium* L3, the serum was pre-incubated with bacteria at a concentration of 10^9^ CFU/ml and incubated at 37°C for 60 min with shaking. The bacteria were separated by centrifugation at 2000 *g* for 10 min and the supernatant was analyzed in ELISA with and without SarsS protein treatment. The supernatant was divided into two samples, one of which was supplemented with protein SarsS at a concentration of 4 μg/ml, and the other with an equal volume of PBS. Both samples were incubated at 37°C for 60 min with shaking. Then each sample was analyzed in ELISA as described in paragraph *Western blotting* in 96-well plates with the biofilms of the original (*E. faecium* L3) and modified (L3-SARS) strains on the bottom.

#### Animal Procedures

Female inbreed Balb/c mice were obtained from the laboratory animal nursery “Rappolovo” (Leningrad Region, Russia) and were used in experiments at the age of 10 weeks. Female inbreed rabbits (2.5 kg weight) were obtained from the laboratory animal nursery “Rappolovo” (Leningrad Region, Russia). Mice and rabbits were housed under standard laboratory conditions with food and water ad libitum. The experiments were developed in accordance with EU Directive 2010/63/EU for animal experiments, approved and carried out according to guidelines and under the supervision of the local biomedical ethics committee (minutes of the meeting 1/21 dated January 28, 2021).

### Statistical Analyses

The results are presented as the mean ± standard deviation of the mean (SEM). The statistical data analysis was performed using the Student’s t-test and *p*-values <0.05 were considered as statistically significant. Statistical data processing was performed using the Statistica 12.0 software package (StatSoft, Inc. Tulsa, Oklahoma). For statistical analysis, antibody titers were expressed as the log10 of the final reciprocal dilution.

### Bioinformatics Analyses

DNA and putative protein analysis were performed employing BLAST NCBI (http://blast.ncbi.nlm.nih.gov/Blast.cgi) and ExPASy (http://www.expasy.org program) packages available in public domains. DNA primer design was accomplished by Primer 3.0 computer program. Protein sequence analysis for the presence of the B-cell and T-cell epitopes was preformed employing IDEB (Immune Epitope Database) analysis and data base resource tool (https://www.iedb.org/).

### Electron Microscopy

Immunoelectron microscopy was aimed to study the structure of *E. faecium* L3 pili with expression of viral proteins. Bacteria were grown in LB (lysogeny broth, VWR Life Science Products Amresco, Solon, United States) medium at 37°C for 18 h. *E. faecium* L3-SARS was cultivated with 5 μg/ml of erythromycin. Bacteria were washed three times in PBS by centrifugation at 3,500 rpm for 20 min and suspended in 0.1 M NaCl. There was 10x concentrate used for immune-electron microscopy. The source of primary antibodies was human polyclonal serum containing IgG specific for S1 of Sars-Cov-2 which were previously adsorbed twice on pure L3 culture in a ratio of 1:10 in order to avoid nonspecific binding. Immunogold labeling was performed using goat IgG conjugated to 18 nm gold particles (1 mg/ml; Jackson ImmunoResearch Laboratories, West Grove, United States). The bacterial culture samples were applied to the grids for transmission electron microscopy (FF400-AU Formvar Support Film 5–6 nm thick on Square, 400 mesh Gold Grid, Electron Microscopy Science, Hatfield, PA) by the method for liquid suspensions (“drop on the grid”) followed by incubation for 2 min at room temperature. Then the grids were fixed for 1 min in 2.5% paraformaldehyde in PBS. Blocking was performed with 0.1% gelatin in PBS for 1 h. Primary antibodies were diluted in 2% BSA/PBS, incubation lasted for 1 h. Secondary antibodies were diluted 1:20 in 2% BSA/PBS, and the samples were incubated for 1 h. For contrasting, 1% uranyl acetate was used in a drop of 20 μL (20 s). To compact the resulting films, they were covered with a layer of nanocarbon. Electron microscopy was performed on a JEM-2100 transmission electron microscope (JEOL, Tokyo, Japan). Photos were taken with digital cameras: bottom port - Gatan Ultrascan 4,000 16 Mpix, 4 × 4 K, 16 bit; side port - Gatan Erlangshen 500 1.4 Mpix, 1.3 × 1 K, 12 bit, 15 fps (Gatan, Pleasanton, United States).

## Results

### Bioinformatics Analysis

**FIGURE 1:**
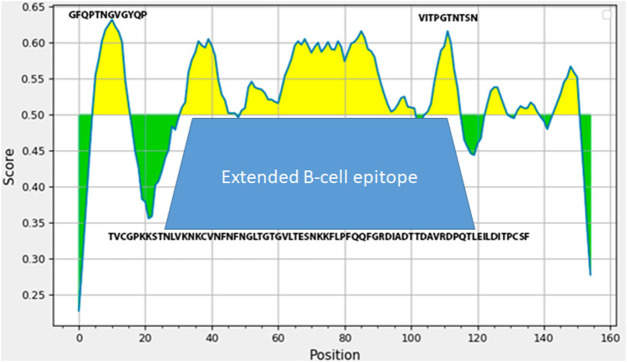
Analysis of SARS-Cov-2 S-protein fragment (amino acids 496-646 in SARS-Cov-2 S protein sequence) employing IDEB (Immune Epitope Database). The amino-acids sequence underneath the trapezoid figure represents large 53 AA immunogenic domain of SARS-Cov-2.

The technology of making live enterococcal vaccine a candidate requires insertion of the heterologous gene fragment in the range of about 500 bp in order to generate a chimeric protein capable of assembly into pili on the surface of bacteria. In order to select the region of the spike protein gene of that size encoding for immunogenic S-protein domains, we have used several protein analysis tools including ExPASy and IDEB on-line platforms. Bioinformatics analysis of S protein sequence obtained from NCBI database revealed an extended B-cell linear epitope of 52 amino acids NFNGLTGTGVLTESNKKFLPFQQFGRDIADTTDAVRDPQTLEILDITPCSF in the area of close proximity of RBD domain with several shorter epitopes TVCGPKKSTNLVKNKCV, and VITPGTNTSN are in the same region (Figure 1). This very area of S protein contained several putative T-cell epitopes (data not shown). The DNA sequence corresponding to this S protein area was synthesized and used to make the expression plasmid *pQE-sarsS* and suicidal plasmid *pentF-sarsS* which was constructed for insertion into the bacterial genome (Supplementary Figure S1). The sequence was deposited into GeneBank (Spike_SARS_incertion OL447006-1 https://www.ncbi.nlm.nih.gov/nuccore/OL447006.1/).

### Development and Analysis of Recombinant Protein SarsS

Construction of the recombinant plasmid pQE-sarsS was performed as described in *Development and cloning of a fusion gene entF- sarsS*. Recombinant DNA was obtained after cloning of the selected frаgment of SARS-Cov-2 DNA into expression plasmid pQE-30. Resultant plasmid designated as *sarsS* was used for transformation of the producer strain of *E. coli* M15 with production of recombinant protein SarsS.

The molecular weight of recombinant protein SarsS was determined as (24, 5 ± 0.5) kDa. (Supplementary Figure S2A). This size corresponded with the predicted size of the amino acid sequence of 168 amino acid residues belonging to SARS-CoV-2 linked to 21 amino acid residues encoded by the vector plasmid pQE-30. The analysis of the amino acid sequence of recombinant protein SarsS was also confirmed by MALDI TOF/TOF.

Purified recombinant protein SarsS was also able to bind IgG obtained from the patients with Covid-19 (Supplementary Figure S2B). The recombinant protein SarsS was subjected to polyacrylamide gel electrophoresis (PAAG) and stained with (Supplementary Figure S2A) Amido Black; the same protein was transferred to nitrocellulose membrane (Supplementary Figure S2B) and blotted with IgG obtained from COVID-19 patients as described in *Immunization Schemes and Antibodies* and *Western Blotting*. The molecular weight of the protein bands stained in immunoblotting coincided with the weight of protein SarsS1 and its dimer and was approximately equal to 25 and 50 kDa.

**FIGURE 2:**
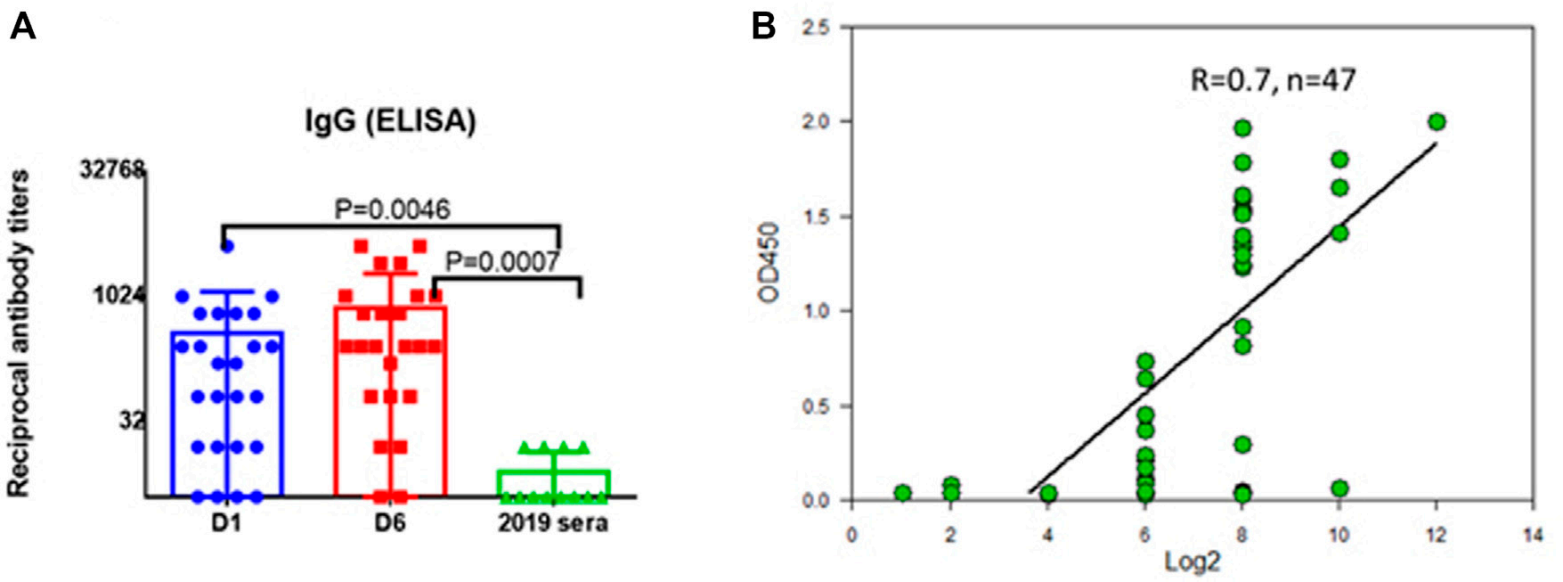
Serum IgG to recombinant SarsS-protein in patients with confirmed SARS-CoV-2 infection. **(A)** D1—first day of hospital stay (n = 28), D6—sixth day of hospital stay (n = 28), 2019 sera were obtained from the patients examined in 2019 (n = 14). **(B)** 47 serum samples from patients with confirmed SARS-CoV-2 infection were studied using a commercial kit for detecting IgG antibodies to coronavirus “SARS-Cov-2 IgG Screen” (Imbian, Russia) in comparison with recombinant S1 protein. A high level of correlation was shown (Spearman’s rank correlation coefficient = 0.7, n = 47) when detecting serum antibodies to SARS-CoV-2 using a recombinant protein and using a commercial kit. A high level of correlation was shown when serum IgG were detected using “SARS-Cov-2 IgG Screen” kit or recombinant S-protein, Spearmen r = 0.7. The positive value according to the screen data corresponded to 1:256 antibody titers obtained using S-protein.

The correspondence of the recombinant protein to its natural version was investigated by immunoblotting with sera of people who had undergone coronavirus infection. The recombinant protein SarsS was found to interact with antibodies in human immune serum obtained from the COVID-19 patients employing ELISA (Figure 2A). Results of detection of the antibodies against SARS-Cov-2 with SarsS protein nicely correlated with the results of the assay with commercial coronavirus detection system “SARS-Cov-2 IgG Screen” (Imbian, Russia) (Figure 2B).

**FIGURE 3:**
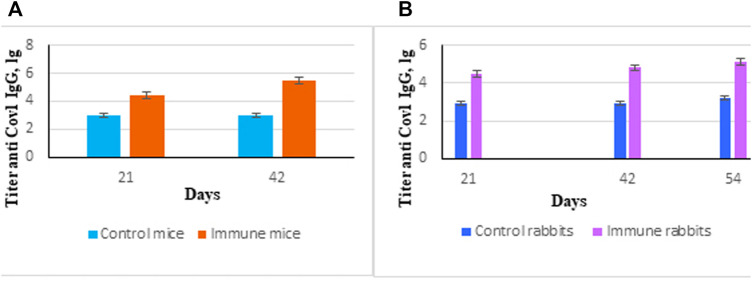
Immune response induced by immunization of animals with recombinant protein SarsS. **(A)** Mice (n = 10) were immunized twice subcutaneously with the recombinant protein SarsS together with aluminum hydroxide as adjuvant. Control mice (n = 10) received aluminum hydrochloride as an adjuvant. Antibody titer was determined by ELISA as described in *Western Blotting*. **(B)** Rabbits (n = 3) were immunized three times subcutaneously with the recombinant protein SarsS together with aluminum hydroxide as vaccine adjuvant. Control rabbits (n = 2) received aluminum hydrochloride as an adjuvant. Antibody titer was determined by ELISA as described in *ELISA Assay*. Reciprocal antibody titers were characterized as log10 (ELISA) and presented as mean ± SEM. The *p*-values are obtained after comparison with control animals. Data were analyzed using the Student’s t-test * - *p* < 0.05.

Purified protein SarsS was used for immunization of mice and rabbits as described in the *Materials and Methods* section. Specific IgG started to appear on Day 21 with a small additional increase at Day 42 (Figures 3A,B). This protein was used in ELISA as an antigen to determine the level of antibodies in serum and nasal lavages as well as for subcutaneous immunization to obtain specific hyperimmune serum. Twofold subcutaneous immunization of mice or threefold immunization of rabbits with protein SarsS in the presence of an adjuvant showed a significant amount of the S-protein specific IgG in blood serum of both types of animals. This data demonstrated that SarsS protein was able to generate specific SARS-Cov-2 immune response after subcutaneous immunization which was reflected in IgG production and immunogenicity of the recombinant protein SarsS.

However, there was only a little increase of S specific IgA in blood serum of mice which might reflect the way of antigen delivery (Supplementary Figure S3).

### Development and Cloning the Fusion Gene *entF-sarsS*

Recombinant plasmid DNA *pentF-sarsS* was constructed as described in *Development and cloning of a fusion gene entF- sarsS*.

The plasmid was sequenced employing DNA primers presented in Table 1. Results of DNA sequencing demonstrated that *SarsS* was inserted in frame with the sequence of *E. faecium* MPP gene. Thus, p*entF-sarsS* plasmid DNA contained inserts of the *sarsS* gene fragment and the flanking fragments of the MPP gene necessary for recombination into the *E. faecium* L3 genome.

**FIGURE 4:**
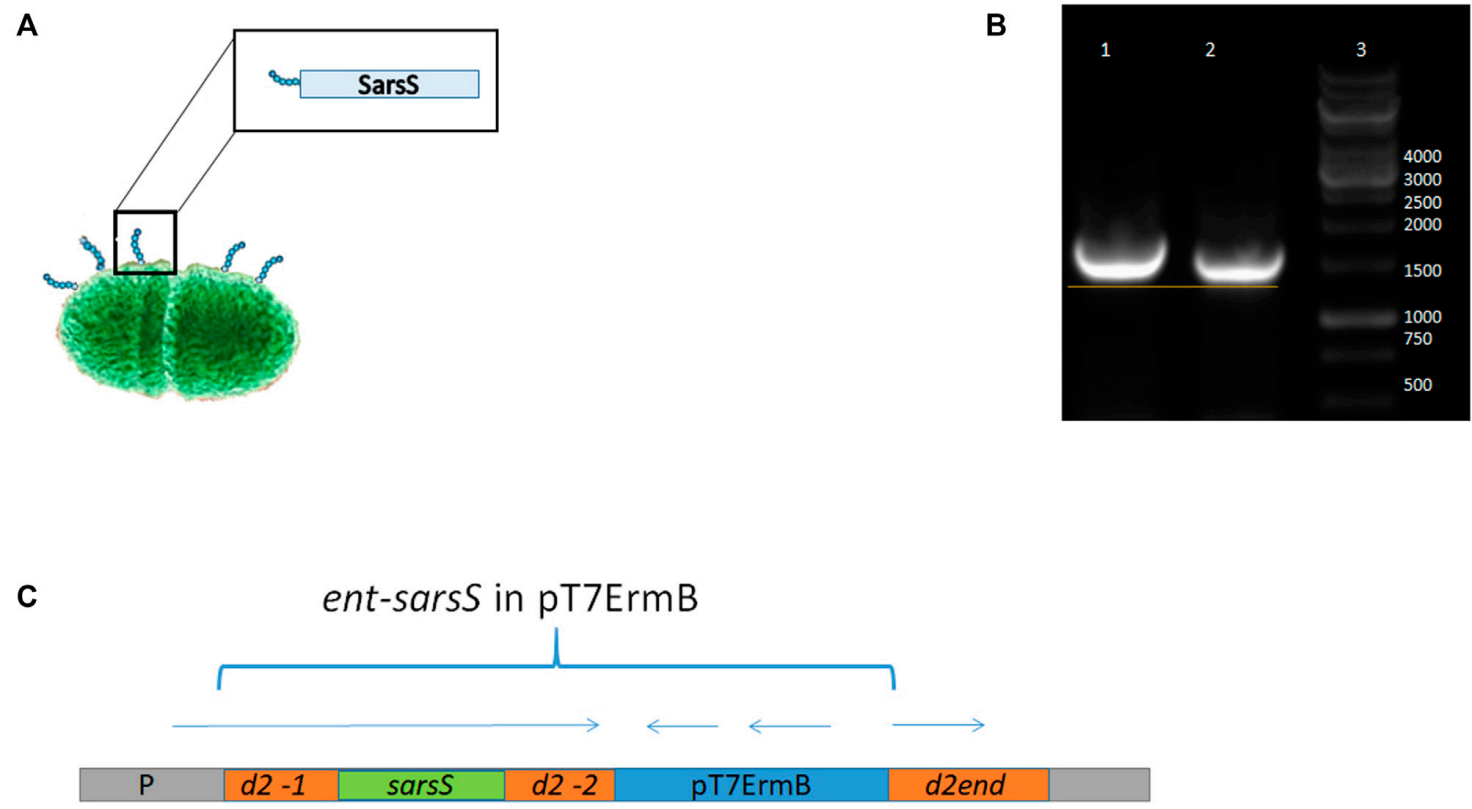
Schematic representation of SARS-Cov-2 DNA fragment integration in enterococcal pili gene. **(A)** Putative enterococcal cell with pili protein containing S1 protein fragment. **(B)** PCR with DNA primers flanking the insert in the strain L3-SARS relatively to the appropriate region of the strain-recipient *E. faecium* L3. It can be seen that in the recombinant strain the DNA insert is slightly larger due to the insertion of SARS-Cov-2 DNA. **(C)** Scheme of the genomic region of enterococci after the integration of the plasmid *pentF-sarsS* into *E. faecium* L3 genome.

The scheme of enterococcal clone design is presented in Figure 4.

### Electro Transformation of Enterococci

Fourteen transformants were obtained after electroporation of enterococci and delivering of the integrative plasmid *pentF-sarsS*. All of them were tested in a PCR reaction with primers K1 and K2. To prove the integration of the *pentF-sarsS* plasmid DNA into the *Enterococcus* genome, the DNA isolated from all clones was amplified with primers B1 and K2. B1 is a primer for the *Enterococcus* MPP gene; K2 is a primer for the insert. Only two clones in the PCR reaction showed the presence of a necessary fragment, the size of which was equal to the sequence between primers B1 and K2 (Table 1). DNA sequencing confirmed that integration of the plasmid *pentF-sarsS* took place exactly as it was originally designed–in the homology region of MPP d2-1 which is located upstream *SarsS* (Supplementary Figure S4). One of the *Enterococcus faecium* L3 clones having the SarS protein gene fragment of SARS-CoV-2 in frame with enterococcal MPP protein gene was selected as a vaccine strain for further research and designated as L3-SARS.

### Study of the Expression of DNA Fragment of the Spike Protein S in the Composition of L3-SARS

We studied the expression of the inserted viral gene fragment *sarsS* at the stage of mRNA synthesis, SarsS protein production, and its localization in a bacterial cell.

#### mRNA Expression

**FIGURE 5:**
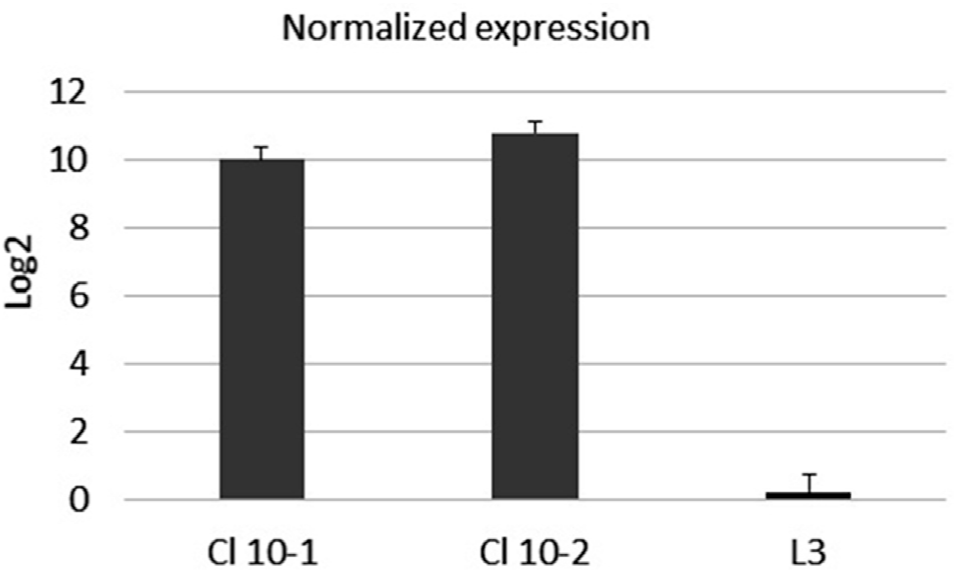
The mRNA expression level of SarsS-protein gene fragment in the culture of *E. faecium* L3. Data were analyzed using the comparative threshold cycles (ΔΔCt) method, normalized to D-alanine-D-alanine ligase gene of L3, and presented as the fold changes in gene expression of S-protein modified L3 relative to the pure L3. Black bars represent the averaged data obtained based on two L3-SARS cultures, the bar at the right represents the averaged data obtained with pure *E. faecium* L3. The average Ct values were 13.5 when RNA from modified L3-SARS or *E. faecium* L3 was amplified using primers to the D-alanine-D-alanine ligase as a housekeeping gene. When using primers specific to S-protein, RNA isolated from modified *E. faecium* L3 cultures gave average Ct values of 28.5, and for pure *E. faecium* L3 average Ct values were more than 40 which indicated the absence of amplification.

The expression of mRNA in real-time PCR with reverse transcriptase (rRT-PCR) was examined with primers specific for the S-protein, according to the procedure described in *Transcription of the SarsS protein gene fragment inserted in bacterial DNA*. To confirm the expression of the inserted *sarsS* gene fragment in bacterial DNA, we studied the expression of mRNA using real-time reverse transcriptase PCR (rRT-PCR) with *SarsS* specific primers K1 и K2. (Table 1). Results of rRT-PCR demonstrated a dramatic increase of amplification of the *SarsS* sequence relative to the control (Figure 5).

These results assured that *sarsS* DNA in *E. faecium* L3 genome is transcribed together with the target gene in *Enterococcal faecium* genome.

#### SarsS Protein Expression in L3-SARS

In order to prove that the spike protein gene fragment fused to the enterococcal MPP gene is properly translated in the strain L3-SARS we lysed the recombinant bacteria and tested the cell lysate in an immune blotting procedure. For this purpose both modified and original *E. faecium* L3 strains were grown overnight, washed, disrupted, and tested in 10% PAAG and Western blotting with S1-specific monoclonal antibodies (Supplementary Figure S5). Only L3-SARS cells lysate generated a band around 80 KDa which was binding the monoclonal antibodies. This band corresponded to the expected size of the MPP protein (Supplementary Figure S5) which was slightly larger than the original MPP because the coding area of the chimeric gene exceeded the size of the original gene of the major pili protein. As expected, monoclonal antibodies against S protein did not bind to *E. faecium* L3.

#### Detection of S Protein Antigenic Epitopes on the Surface of Enterococci

**FIGURE 6:**
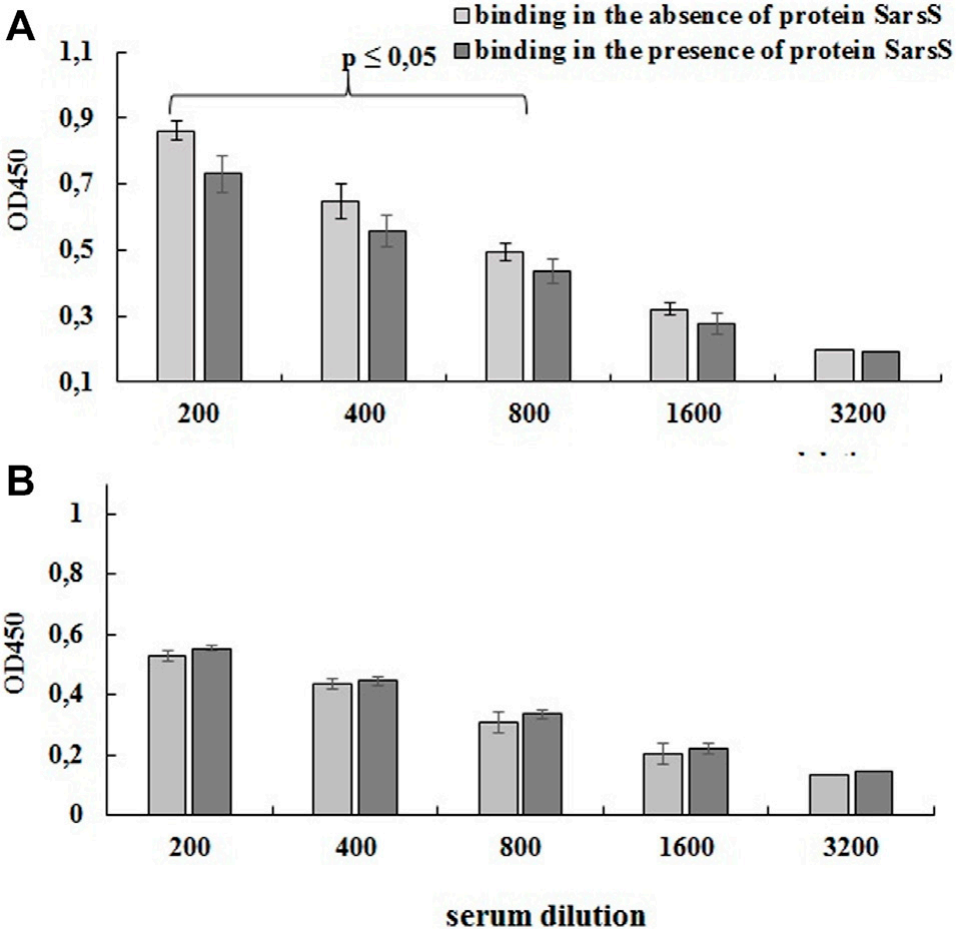
Detection of protein SarsS on the surface *of E. faecium* L3 by ELISA. A serum of patients recovered from SARS-CoV-2 was analyzed by competitive ELISA in the 96-well plates covered with L3-SARS **(A)** and *E. faecium* L3 **(B)**. The results allowed evaluating the effect of SARS-Cov-2 specific serum to bind modified *Enterococcus* strain after the preliminary incubation with recombinant SarsS protein. The figure shows the data of one of the three independent experiments, the conclusions from which completely coincided. Each point is the average of four measurements. Data presented as mean ± SEM. Data were analyzed using the Student’s t-test.

For this purpose, bacterial cells on the bottom of the immunological 96 wells plates were probed with the human serum which was preliminarily adsorbed with purified SarS recombinant protein as described in Section 2.3.4. As shown on Figure 6A preliminary incubation of the serum with recombinant protein SarsS significantly decreased the level of the binding of antibodies with L3-SARS. On the contrary, when *E. faecium* L3 was tested in the same experiment we could not determine any difference (Figure 6B). This data show that SARS-CoV-2 specific antigens are expressed on the surface of recombinant bacteria L3-SARS.

**FIGURE 7:**
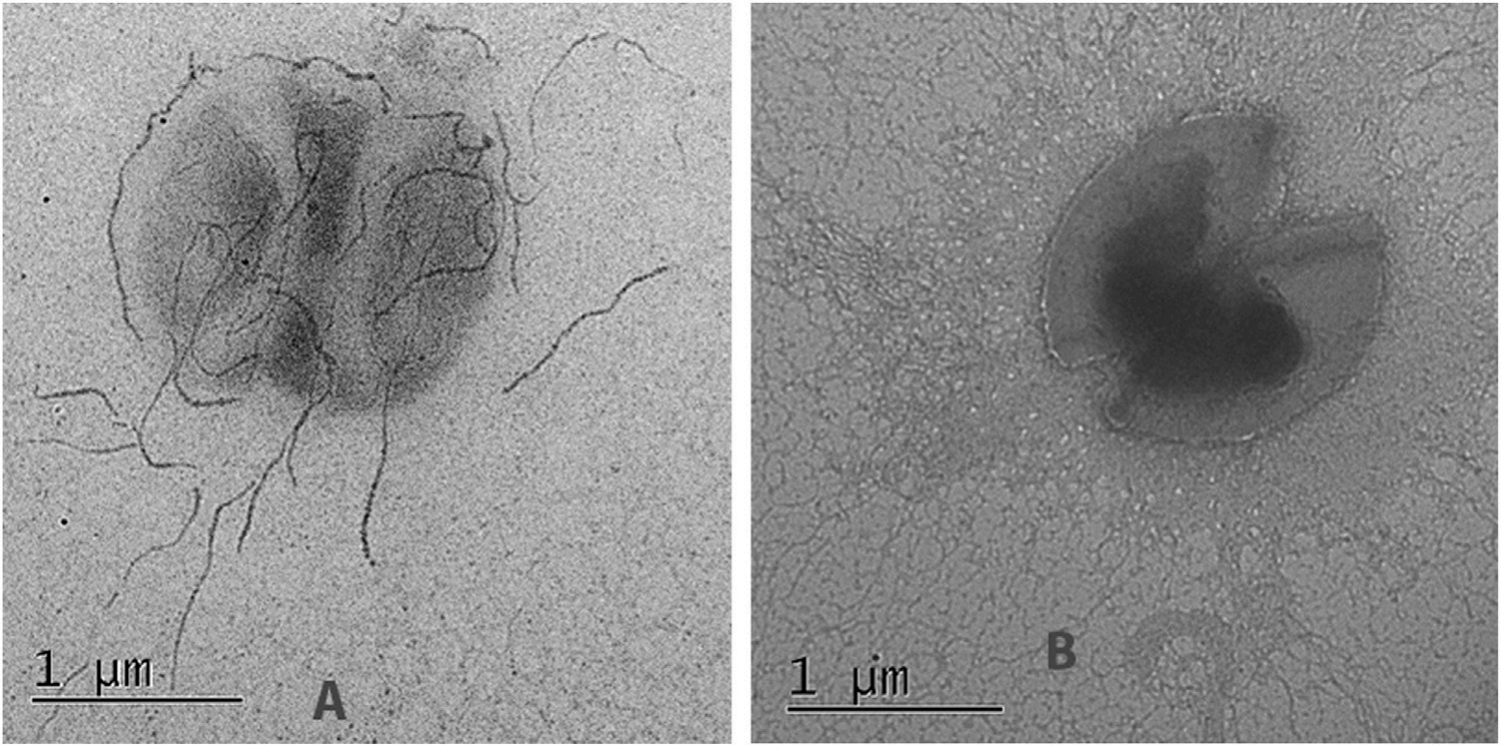
Immunoelectron microscopy of the original (*E, faecium* L3) and genetically modified strain (L3-SARS) after detection with polyclonal serum from patients with COVID-19 with the following immunogold labeled goat IgG conjugated to 18 nm gold particles as described in Section 2.8. **(A)** strain L3-SARS. **(B)** *E. faecium* L3.

Electron microscopy of the strain L3-SARS depicted numerous chains of more than 10 molecules interacting with the gold label. Interestingly some of the label accumulates just on the cell surface depicting differential expression of the pili protein changed by genetic manipulations (Figure 7A). As expected*, E, faecium* L3 was free from the specific interaction with IgG from COVID-19 patients (Figure 7B). Immune electron microscopy data shows that a fragment of spike protein from SARS-Cov-2 not only expresses on the surface of enterococcal recombinant strain but is also capable of being part of a properly assembled enterococcal pili. This finding makes the strain L3-SARS an interesting vaccine candidate due to the easy access of pili to the host immune system.

## Discussion

Delivery of the antigens to the mucous membranes as part of the viral or bacterial vector is a reasonable approach, since it overcomes several problems inherent in parenteral vaccines. Intranasal or oral delivery of the antigen is convenient, non-traumatic, and economical. The oral antigen delivery system usually is able to stimulate an intense mucosal immune response. This distinguishes the mucosal route from parenteral vaccination (Taghinezhad-S et al., 2021).

Various pre-clinical and clinical studies provided the evidence that oral immunization offers several advantages over other ways of immunizations. This includes better stimulation of gut-associated lymphoid tissue (GALT), enhanced production of anti-viral IgA, effective induction of mucosal immune responses, decreased risk of contamination, cost-effectiveness, and easy self-administration or administration to animals (Qiao, et al., 2009; Wang et al., 2012; Mohseni et al., 2020). Antigen production by live probiotic bacteria capable of multiplying in the gut and affecting larger mucosal area for a prolonged period of time is an additional advantage of probiotic vaccines.

All these data provide a substantial amount of information advocating the usage of probiotic vaccines as defense against viral pathogens. What is important, oral vaccination, compared to the nasal route, can significantly increase DC activation, specific IgA production, CD8^+^ T-cell induction, and cross-protection against viral challenge *in vivo* (Yang et al., 2016). Additionally, *in vivo* studies proved that oral intake of recombinant LAB can provide higher neutralizing antibody activities compared to intraperitoneal injection (Sim et al., 2008; Tang and Li, 2009).

It is also important to notice that the state and condition of microbiota provide an additional defense against the viral pathogens on mucosal surfaces which make probiotic bacteria extremely useful as factors positively influencing the microenvironment. Clinical studies and human trials suggest that several probiotic strains including L. rhamnosus GG, L. casei, L. plantarum, L. casei strain Shirota, B. lactis Bb-12, and B. longum were able to reduce the prevalence of upper respiratory infections, common cold, flu-like symptoms, and antibiotic-associated diarrhea by 40–70% (Rautava et al., 2009; Smith et al.,. 2013; King et al., 2019). Furthermore, probiotic strains such as L. reuteri ATCC 55730, L. paracasei, L. casei 431, L. fermentum PCC, and B. infantis 35,624 were pivotal in producing immunomodulatory responses during various infections (Oliva et al., 2012; Pu et al., 2017; Zhang et al., 2018; Villena et al., 2021).

The hostile environment of the gastrointestinal tract, which includes the stomach extreme pH and the intestinal protease-rich environments, can severely affect the immunogenicity of ingested antigens. These characteristics have made the generation of efficient oral vaccines extremely challenging, due to the difficulty of finding appropriate antigen delivery systems and adjuvants that efficiently stimulate mucosal immunity.

Integration of vaccine antigens into the genome of probiotic microorganisms provides additional advantages to probiotic vaccines. The beneficial effects of the administration of probiotics complement the effect of specific immune stimulation and enhance the mucosal immunity (Singh and Rao 2021). The aim of the present study was to construct a novel vaccine candidate in a form of live bacterial vaccine with a gene fragment encoding to evaluate the S-specific immune response after the oral administration of a live vaccine. For this purpose, it was necessary to select a bacterial vector, viral fragment with immunogenic features, and to generate bacteria, capable of expressing SARS-Cov-2 epitopes on the surface.

Previously we developed and successfully used a method for inserting gene fragments of pathogenic streptococci into the *E. faecium* L3 genome to obtain live antibacterial vaccines (Gupalova et al., 2018; Gupalova et al., 2019). *E. faecium* L3 is a well-studied probiotic bacteria with good clinical evidence of safety and usage in the case of chronic gastro-intestinal diseases, *H. pylori* infections, and multiple sclerosis (Lo Skiavo et al., 2013; Baryshnikova et al., 2015; Abdurasulova et al., 2016; Suvorov, 2020).

The method designed to incorporate streptococcal DNA fragments into the enterococcal gene encoding the major pili protein and thus express the foreign protein on the surface of the probiotic bacterium rely on the permanent integration of the suicidal plasmid into bacterial genome. It is well established that Gram-positive bacteria, including *Streptococcus pneumoniae* and *E. faecium,* assemble long filamentous pili on their surface through which they adhere to host cells (Nallapareddy et al., 2006; Spraggon et al., 2010; Khare and Narayana, 2017).

For example, pneumococcal pili are formed by a backbone, consisting of the repetition of the major component RrgB (or MPP in *Enterococcus*)—main repetitive structural domain of the native pilus with LPXTG motive in C terminus of the protein. This motive is necessary for the attachment of the protein subunit with N terminus of the next molecule in the chain of the pili. In the present study, part of the MPP gene with the d2 domain was replaced with a part of the S glycoprotein (spike) if modification of the central part of the gene will not interfere with the protein chain assembly. The SARS-Cov-2 S glycoprotein (∼141 kDa and ∼1,270 amino acids) is responsible for the viral attachment to the human cell receptor ACE-2. We have selected an immunogenic part of the protein, which is located in an area of close proximity with ACE-2 binding domain and inserted 501 bp DNA fragment encoding for this region into the probiotic genome. Analysis of the resultant recombinant enterococcal strain by PCR and following DNA sequencing revealed the presence of the spike DNA sequence in enterococcal genome (Supplementary Figure S4). The strain, selected on the agar plates with antibiotic, was highly stable and did not lose the insert after 48 generations in media without antibiotic. In the organism of mice after feeding it stayed more than a week which proved that the strain L3-SARS was able to multiply in the animal gut (data not shown).

The spike gene fragment cloned in the expression plasmid was successfully tested for the ability to induce specific immunity in two different types of animals–mice and rabbits. In addition, the protein SarsS was specifically interacting with human IgG of the COVID-19 patients. This DNA sequence cloned in probiotic genome in frame with the pili protein gene directed synthesis of S-specific mRNA and the appearance of the surface protein capable of binding to the monoclonal antibodies against S-protein epitopes. Surface display testing of the resultant enterococcal strain L3-SARS revealed that the recombinant bacteria possessed the ability to specifically bind IgG from COVID-19 patients. Interestingly the cells of the original enterococcal strain *E. faecium* L3 were also binding human immunoglobulins. It is known that the natural immune response of mammals to microbiota includes T-independent induction of polyspecific antibodies, which are present in blood serum and act as a regulator of commensal microbiota including enterococci (Pabst et al., 2016; Zeng et al., 2016).

After the co-incubation of the antibodies with the SarsS protein, their binding to the *E. faecium* L3 and L3-SARS revealed significant differences. Indeed, pre-incubation with SarsS protein significantly reduced the binding of immune serum IgG to the L3-SARS surface relatively to control (Figure 6).

SARS-Cov-2 specific antigens on the surface of L3-SARS were determined by electron microscopy (Figure 7A) which demonstrated the proper assembly of the chimeric pili molecules on the surface of bacteria.

Taken together the results of the study allow concluding that selected fragments of SARS-Cov-2 DNA were able to direct synthesis of immunogenic protein that was expressed by the strain of *E. faecium*. Further experiments of the strain L3-SARS regarding its ability to generate the specific humoral and T-cell response are required for evaluating the possibility using this strain as a candidate for oral vaccine against SARS-Cov-2.

## Conclusion

The aim of the study was to generate a novel probiotic vaccine candidate for mucosal immunization against SARS-Cov-2. The fragment of SARS-Cov-2 DNA encoding for S1 protein was cloned in the *E. coli* expression vector, isolated as recombinant protein, and tested for immunogenicity in mice and rabbits. This DNA fragment was also inserted in *E. faecium* probiotic genome. The resultant strain L3-SARS was expressing SARS-Cov-2 specific epitopes on the surface able to react with specific IgG from the COVID-19 patients and S1 specific monoclonal antibodies. Further experiments of the strain regarding its ability to generate the specific humoral immune response, T-cell response, and anti-SARS-Cov-2 protection will follow.

## Data Availability

The original contributions presented in the study are publicly available. This data can be found here: https://www.ncbi.nlm.nih.gov/nuccore/OL447006.1/.

## Supplementary Material

Supplementary Figure S1 DNA sequence of the *sarsS* gene used for cloning.

Supplementary Figure S2 PAGE analysis of recombinant protein SarsS and immunoblotting with antibodies from COVID-19 patients. The recombinant protein SarsS was subjected to polyacrylamide gel electrophoresis (PAGE) and stained with (lane A) Amido Black; the same protein was transferred to nitrocellulose membrane (lane B) and blotted with IgG obtained from COVID-19 patients as described in *Immunization schemes and antibodies* and *Western blotting*. 1) SarsS protein. 2) Protein molecular weight marker. 3 –Immunoblotting of SarsS being transferred on the nitrocellulose with IgG obtained from COVID-19 patients.

Supplementary Figure S3 Immune response to subcutaneous immunization of mice with protein SarsS on Day 42 after the first injection.

Supplementary Figure S4 Chromosomal DNA region of *Enterococcus* with the nucleotide sequence of the integrated plasmid DNA *pentF-sarsS .*DNA primers B1 and K2 used for sequencing are highlighted in bold.

Supplementary Figure S5 Detection of protein SarsS in lysate of *E. faecium* L3 by Western blotting. Enterococcal proteins were separated by electrophoresis in 10% PAGE, transferred to a nitrocellulose membrane and blotted with commercial monoclonal antibody (AtaGenix Laboratories, Wuhan, China), specific to S1 protein of SARS-CoV-2.
