# Supplementary figures and images for: Construction of the Enterococcal Strain Expressing Immunogenic Fragment of SARS-Cov-2 Virus
Source: Front Pharmacol. 2022 Jan 5;12:807256. doi: 10.3389/fphar.2021.807256 (PMC8823703; doi:10.3389/fphar.2021.807256)

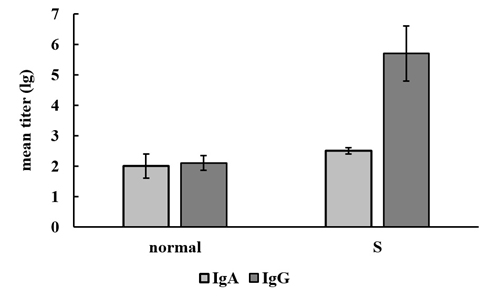

Supplement: Supplementary file 1 [file Image3.JPEG]

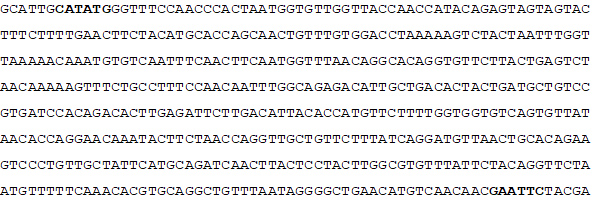

Supplement: Supplementary file 2 [file Image1.JPEG]

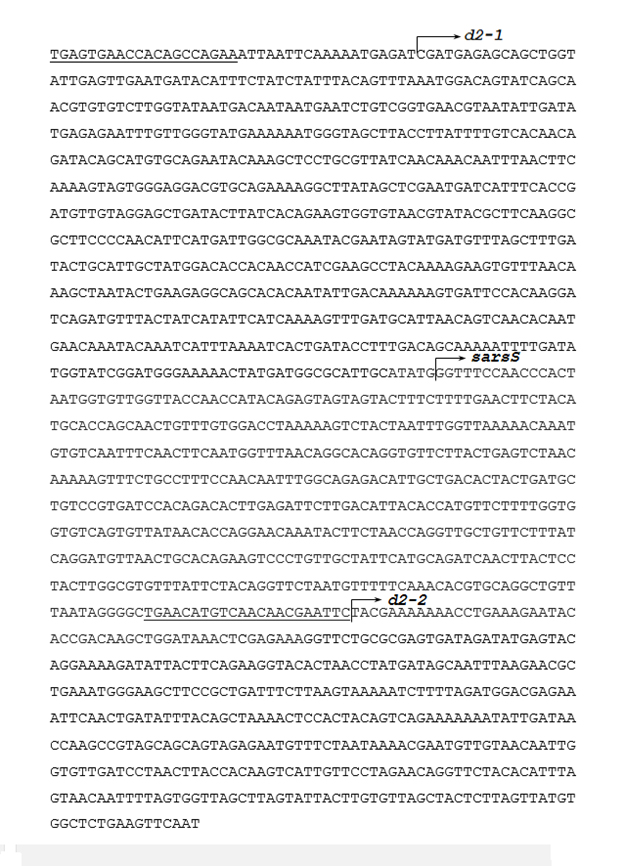

Supplement: Supplementary file 3 [file Image4.JPEG]

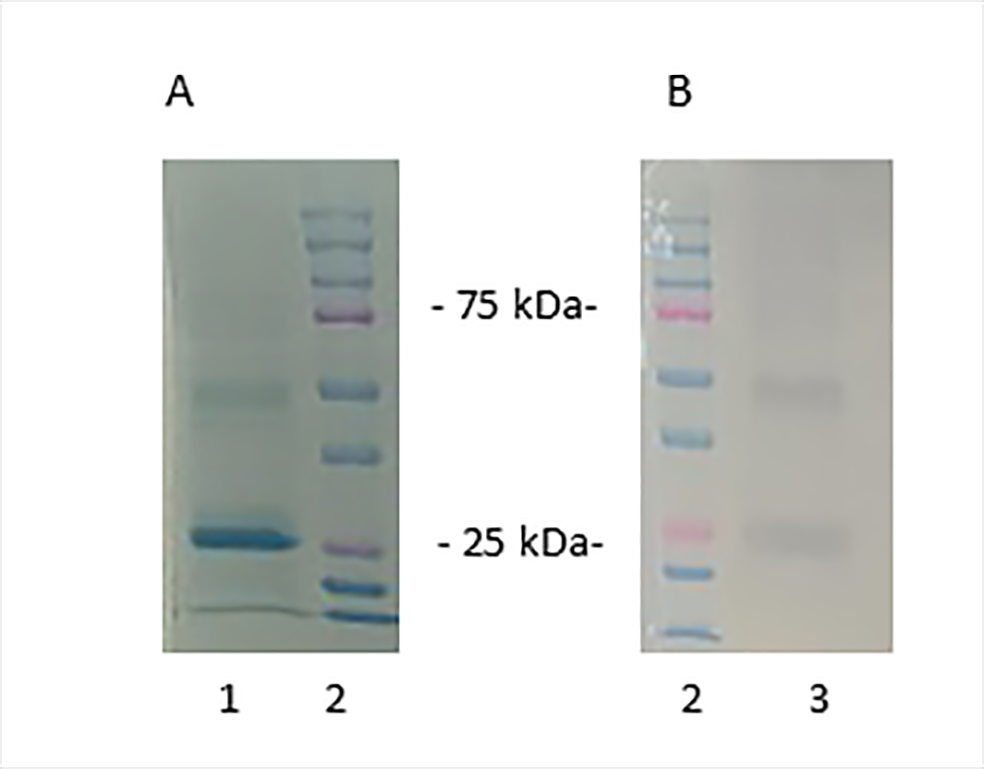

Supplement: Supplementary file 4 [file Image2.TIF]

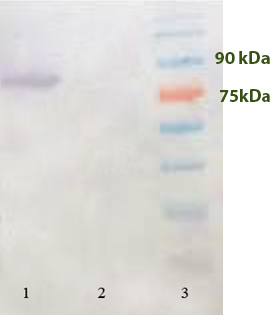

Supplement: Supplementary file 5 [file Image5.JPEG]
